# Supplementary material for: Different Mechanisms of Action of Regorafenib and Lenvatinib on Toll-Like Receptor-Signaling Pathways in Human Hepatoma Cell Lines
Source: Int J Mol Sci. 2020 May 9;21(9):3349. doi: 10.3390/ijms21093349 (PMC7246870; doi:10.3390/ijms21093349)
Supplement: Supplementary file 1 [file ijms-21-03349-s001.pdf]

Table S1 The 84 genes associated with Toll-like receptor signaling pathway assessed by real-time RT-PCR, and gene expression of regorafenib (vs. lenvatinib) in the present study.

(A) Toll-like receptors		Regorafenib (vs. Lenvatinib) (Fold Change)	
Symbol	Name	Huh7	HepG2
CD180 (LY64)	CD180 molecule	1.65	**0.25**
SIGIRR	Single immunoglobulin and Toll-interleukin 1 receptor (TIR) domain	1.31	0.71
TLR1	Toll-like receptor 1	**1.90**	0.63
TLR2	Toll-like receptor 2	0.70	**4.90**
TLR3	Toll-like receptor 3	**1.39**	**0.64**
TLR4	Toll-like receptor 4	**0.22**	**0.32**
TLR5	Toll-like receptor 5	1.24	0.72
TLR6	Toll-like receptor 6	0.59	0.63
TLR7	Toll-like receptor 7	1.53	**4.90**
TLR8	Toll-like receptor 8	1.32	**4.90**
TLR9	Toll-like receptor 9	1.48	0.80
TLR10	Toll-like receptor 10	**1.97**	**2.39**

Bold, p <0.05; red color, p<0.05 with both cell lines and same vector.

(B) Pathogen-specific responses: Bacterial		Regorafenib (vs. Lenvatinib) (Fold Change)	
Symbol	Name	Huh7	HepG2
CCL2 (MCP-1)	Chemokine (C-C motif) ligand 2	**1.97**	**0.31**
CD14	CD14 molecule	**0.73**	**0.25**
CD180 (LY64)	CD180 molecule	1.65	**0.25**
FOS	FBJ murine osteosarcoma viral oncogene homolog	**0.05**	0.64
HRAS	v-Ha-ras Harvey rat sarcoma viral oncogene homolog	1.04	0.93
IL10	Interleukin 10	1.70	1.29

IL12A	Interleukin 12A	**0.59**	1.04
IL1B	Interleukin 1, beta	**0.28**	**0.48**
IL6	Interleukin 6 (interferon, beta 2)	3.97	**0.24**
IL8	Interleukin 8	**0.46**	**0.28**
IRAK1	Interleukin-1 receptor-associated kinase 1	1.05	1.12
HMGB1	High mobility group box 1	0.93	1.01
HSPA1A (HSP70 1A)	Heat shock 70kDa protein 1A	0.99	**0.61**
JUN	Jun proto-oncogene	1.75	0.60
LTA (TNFB)	Lymphotoxin alpha (TNF superfamily, member 1)	1.11	0.33
LY86 (MD-1)	Lymphocyte antigen 86	**1.93**	**4.90**
LY96	Lymphocyte antigen 96	2.17	0.45
NFKBIA	Nuclear factor of kappa light polypeptide gene enhancer in B-cells inhibitor, alpha	1.23	0.79
PTGS2 (COX2)	Prostaglandin-endoperoxide synthase 2	0.55	0.97
RELA	v-rel reticuloendotheliosis viral oncogene homolog A (avian)	1.02	**0.65**
RIPK2	Receptor-interacting serine-threonine kinase 2	**1.27**	0.98
TLR2	Toll-like receptor 2	0.70	**4.90**
TLR4	Toll-like receptor 4	**0.22**	**0.32**
TLR6	Toll-like receptor 6	0.59	**0.63**
TNFRSF1A	Tumor necrosis factor receptor superfamily, member 1A	0.77	1.31
TICAM1 (TRIF)	Toll-like receptor adaptor molecule 1	1.20	0.96

(C) Pathogen-specific responses: Viral		Regorafenib (vs. Lenvatinib) (Fold Change)	
Symbol	Name	Huh7	HepG2
EIF2AK2 (PKR)	Eukaryotic translation initiation factor 2-alpha kinase 2	1.11	**0.69**
IFNB1	Interferon, beta 1, fibroblast	3.12	2.31
IFNG	Interferon, gamma	**1.97**	**4.90**
IL12A	Interleukin 12A	**0.59**	1.04
IL6	Interleukin 6 (interferon, beta 2)	3.97	**0.24**
IRF3	Interferon regulatory factor 3	1.01	0.90
PRKRA	Protein kinase, interferon-inducible double-stranded RNA dependent activator	1.07	0.86
RELA	v-rel reticuloendotheliosis viral oncogene homolog A (avian)	1.02	**0.65**
TBK1	TRAF member-associated NFKB activator (TANK)-binding kinase 1	1.18	0.83
TLR3	Toll-like receptor 3	**1.39**	**0.64**
TLR7	Toll-like receptor 7	1.53	**4.90**
TLR8	Toll-like receptor 8	1.32	**4.90**
TNF	Tumor necrosis factor	0.96	**0.17**
TICAM1 (TRIF)	Toll-like receptor adaptor molecule 1	1.20	0.96

(D) Pathogen-specific responses: Fungal/Parasitic		Regorafenib (vs. Lenvatinib) (Fold Change)	
Symbol	Name	Huh7	HepG2
CLEC4E	C-type lectin domain family 4, member E	**1.97**	**4.90**
HRAS	v-Ha-ras Harvey rat sarcoma viral oncogene homolog	1.04	0.93
HSPA1A (HSP70 1A)	Heat shock 70kDa protein 1A	0.99	**0.61**

IL8	Interleukin 8	**0.46**	**0.28**
TLR2	Toll-like receptor 2	0.70	**4.90**
TIRAP	Toll-interleukin 1 receptor (TIR) domain containing adaptor protein	1.09	1.00

(E) Toll-like receptor signaling: Negative regulation		Regorafenib (vs. Lenvatinib) (Fold Change)	
Symbol	Name	Huh7	HepG2
SARM1	Sterile alpha and TIR motif containing 1	1.38	**0.50**
SIGIRR	Single immunoglobulin and Toll-interleukin 1 receptor (TIR) domain	1.31	0.71
TOLLIP	Toll interacting protein	1.22	0.97

(F) Toll-like receptor signaling: TICAM1 (TRIF)-dependent (MYD88-independent)		Regorafenib (vs. Lenvatinib) (Fold Change)	
Symbol	Name	Huh7	HepG2
IRF3	Interferon regulatory factor 3	1.01	0.90
MAP3K7 (TAK1)	Mitogen-activated protein kinase kinase kinase 7	0.93	0.80
TAB1	TGF-beta activated kinase 1/MAP3K7 binding protein 1	1.20	0.93
NR2C2	Nuclear receptor superfamily 2, group C, member 2	1.12	**0.64**
PELI1	Pellino E3 ubiquitin protein ligase 1	**0.48**	0.86
TBK1	TRAF member-associated NFKB activator (TANK)-binding kinase 1	1.18	0.83
TICAM2	Toll-like receptor adaptor molecule 2	0.84	**0.62**
TLR3	Toll-like receptor 3	**1.39**	**0.64**

TLR4	Toll-like receptor 4	**0.22**	**0.32**
TRAF6	TNF receptor-associated factor 6, E3 ubiquitin protein ligase	**1.22**	**0.61**
TICAM1 (TRIF)	Toll-like receptor adaptor molecule 1	1.20	0.96

(G) Toll-like receptor signaling: MYD88-dependent		Regorafenib (vs. Lenvatinib) (Fold Change)	
Symbol	Name	Huh7	HepG2
IRAK1	Interleukin-1 receptor-associated kinase 1	1.05	1.12
IRAK2	Interleukin-1 receptor-associated kinase 2	0.75	0.76
MAP3K7 (TAK1)	Mitogen-activated protein kinase kinase kinase 7	0.93	0.80
TAB1	TGF-beta activated kinase 1/MAP3K7 binding protein 1	1.20	0.93
MYD88	Myeloid differential primary response gene (88)	1.09	0.89
NR2C2	Nuclear receptor superfamily 2, group C, member 2	1.12	**0.64**
TIRAP	TIR domain containing adaptor protein	1.09	1.00
TLR1	Toll-like receptor 1	**1.90**	0.63
TLR10	Toll-like receptor 10	**1.97**	**2.39**
TLR2	Toll-like receptor 2	0.70	**4.90**
TLR4	Toll-like receptor 4	**0.22**	**0.32**
TLR5	Toll-like receptor 5	1.24	0.72
TLR6	Toll-like receptor 6	0.59	**0.63**
TLR7	Toll-like receptor 7	1.53	**4.90**
TLR8	Toll-like receptor 8	1.32	**4.90**
TLR9	Toll-like receptor 9	1.48	0.80
TRAF6	TNF receptor-associated factor 6, E3 ubiquitin protein ligase	**1.22**	**0.61**

(H) Downstream pathways and target genes: NF-κB pathway		Regorafenib (vs. Lenvatinib) (Fold Change)	
Symbol	Name	Huh7	HepG2
BTK	Bruton agammaglobulinemia tyrosine kinase	0.33	0.27
CASP8	Caspase 8, apoptosis-related cysteine peptidase	**0.80**	0.70
CHUK (IKKa)	Conserved helix-loop-helix ubiquitous kinase	0.90	**0.64**
ECSIT (SITPEC)	ECSIT homolog (Drosophila)	1.16	0.83
FADD	Fas (TNFRSF6)-associated via death domain	0.81	0.87
IKBKB	Inhibitor of kappa light polypeptide gene enhancer in B-cells, kinase beta	1.14	0.87
IL10	Interleukin 10	1.70	1.29
IL1B	Interleukin 1, beta	**0.28**	**0.48**
IRAK1	Interleukin-1 receptor-associated kinase 1	1.05	1.12
IRAK2	Interleukin-1 receptor-associated kinase 2	0.75	0.76
IRF3	Interferon regulatory factor 3	1.01	0.90
LY96	Lymphocyte antigen 96	2.17	0.45
MAP3K1 (MEKK)	Mitogen-activated protein kinase kinase kinase 1, E3 ubiquitin protein ligase	1.21	**0.65**
MAP3K7	Mitogen-activated protein kinase kinase kinase 7	0.93	0.80
NFKB1	Nuclear factor of kappa light polypeptide gene enhancer in B-cells 1	1.01	0.86
NFKB2	Nuclear factor of kappa light polypeptide gene enhancer in B-cells 2 (p49/p100)	1.12	1
NFKBIA	Nuclear factor of kappa light	1.23	0.79

	polypeptide gene enhancer in B-cells inhibitor, alpha		
NFKBIL1	Nuclear factor of kappa light polypeptide gene enhancer in B-cells inhibitor-like 1	1.29	1.30
NFRKB	Nuclear factor related to kappaB binding protein	0.93	0.95
PPARA	Peroxisome proliferator-activated receptor alpha	1.32	1.03
REL	v-rel reticuloendotheliosis viral oncogene homolog (avian)	0.86	**0.65**
RELA	v-rel reticuloendotheliosis viral oncogene homolog A (avian)	1.02	**0.65**
TNF	Tumor necrosis factor	0.96	**0.17**
TNFRSF1A	Tumor necrosis factor superfamily, member 1A	0.77	1.31
UBE2N	Ubiquitin-conjugating enzyme E2N	**0.77**	0.76

(I) Downstream pathways and target genes: JNK/p38 pathway		Regorafenib (vs. Lenvatinib) (Fold Change)	
Symbol	Name	Huh7	HepG2
ELK1	ELK1, member of ETS oncogene family	0.91	1.26
FOS	FBJ murine osteosarcoma viral oncogene homolog	**0.05**	0.64
IL1B	Interleukin 1, beta	**0.28**	**0.48**
JUN	Jun proto-oncogene	1.75	0.60
MAP2K3 (MEK3)	Mitogen-activated protein kinase kinase 3	1.00	0.91
MAP2K4(JNKK1)	Mitogen-activated protein kinase kinase 4	1.02	0.79
MAP3K1 (MEKK)	Mitogen-activated protein kinase kinase kinase 1, E3 ubiquitin protein ligase	1.21	**0.65**

MAP3K7	Mitogen-activated protein kinase kinase kinase 7	0.93	0.80
MAPK8 (JNK1)	Mitogen-activated protein kinase 8	0.94	**0.67**
MAPK8IP3	Mitogen-activated protein kinase 8 interacting protein 3	1.22	1.00
TNF	Tumor necrosis factor	0.96	**0.17**

(J) Downstream pathways and target genes: JAK/STAT pathway		Regorafenib (vs. Lenvatinib) (Fold Change)	
Symbol	Name	Huh7	HepG2
CCL2 (MCP-1)	Chemokine (C-C motif) ligand 2	**1.97**	**0.31**
CSF2 (GM-CSF)	Colony stimulating factor 2 (granulocyte-macrophage)	0.34	0.70
IFNG	Interferon, gamma	**1.97**	**4.90**
IL12A	Interleukin 12A	**0.59**	1.04
IL2	Interleukin 2	**1.97**	**4.24**
IL6	Interleukin 6	3.97	**0.24**

(K) Downstream pathways and target genes: Interferon regulatory factor (IRF) pathway		Regorafenib (vs. Lenvatinib) (Fold Change)	
Symbol	Name	Huh7	HepG2
CXCL10 (IP10)	Chemokine (C-X-C motif) ligand 10	**3.22**	**0.34**
IFNA1	Interferon, alpha 1	**1.97**	0.64
IFNB1	Interferon, beta 1, fibroblast	3.12	2.31
IFNG	Interferon, gamma	**1.97**	**4.90**
IRF1	Interferon regulatory factor 1	1.40	**0.54**
IRF3	Interferon regulatory factor 3	1.01	0.90
TBK1	TANK-binding kinase 1	1.18	0.83

(L) Downstream pathways and target genes: Cytokine-mediated signaling pathway		Regorafenib (vs. Lenvatinib) (Fold Change)	
Symbol	Name	Huh7	HepG2
CCL2 (MCP-1)	Chemokine (C-C motif) ligand 2	**1.97**	**0.31**
CSF3 (GCSF)	Colony stimulating factor 3 (granulocyte)	1.58	1.50
IL1A	Interleukin 1, alpha	0.58	**0.49**
IL1B	Interleukin 1, beta	**0.28**	**0.48**
IL6	Interleukin 6	3.97	**0.24**
IRAK1	Interleukin-1 receptor-associated kinase 1	1.05	1.12
IRAK2	Interleukin-1 receptor-associated kinase 2	0.75	0.76
RELA	v-rel reticuloendotheliosis viral oncogene homolog A (avian)	1.02	**0.65**
SIGIRR	Single immunoglobulin and Toll-interleukin 1 receptor (TIR) domain	1.31	0.71
TNF	Tumor necrosis factor	0.96	**0.17**
TNFRSF1A	Tumor necrosis factor receptor superfamily, member 1A	0.77	1.31

(M) Regulation of adaptive immunity		Regorafenib (vs. Lenvatinib) (Fold Change)	
Symbol	Name	Huh7	HepG2
CD80	CD80 antigen	1.93	0.50
CD86	CD86 antigen	0.76	**2.09**
HSPD1	Heat shock 60kDa protein1 (chaperonin)	**0.64**	**0.52**
IFNG	Interferon, gamma	**1.97**	**4.90**
IL10	Interleukin 1o	1.70	1.29
IL12A	Interleukin 12A	**0.59**	1.04
IL1B	Interleukin 1, beta	**0.28**	**0.48**
IL2	Interleukin 2	**1.97**	**4.24**

MAP3K7	Mitogen-activated protein kinase kinase kinase 7	0.93	0.80
TRAF6	TNF receptor-associated factor 6, E3 ubiquitin protein ligase	**1.22**	**0.61**

(N) Adaptors & TLR interacting proteins

		Regorafenib (vs. Lenvatinib) (Fold Change)	
Symbol	Name	Huh7	HepG2
BTK	Bruton agammaglobulinemia tyrosine kinase	0.33	0.27
CD14	CD14 molecule	**0.73**	**0.25**
HMGB1	High mobility group box 1	**0.93**	1.01
HRAS	v-Ha-ras Harvey rat sarcoma viral oncogene homolog	1.04	0.93
HSPA1A (HSP70 1A)	Heat shock 70kDa protein 1A	0.99	**0.61**
HSPD1	Heat shock 60kDa protein1 (chaperonin)	**0.64**	**0.52**
LY86 (MD-1)	Lymphocyte antigen 86	**1.93**	**4.90**
LY96 (MD-2)	Lymphocyte antigen 96	2.17	0.45
MAPK8IP3	Mitogen-activated protein kinase 8 interacting protein 3	1.22	1.00
MYD88	Myeloid differentiation primary response gene (88)	1.09	0.89
PELI1	Pellino E3 ubiquitin protein ligase 1	**0.48**	0.86
RIPK2	Receptor-interacting serine-threonine kinase 2	**1.27**	0.98
SARM1	Sterile alpha and TIR motif containing 1	1.38	**0.50**
TICAM1 (TRIF)	Toll-like receptor adaptor molecule 1	1.20	0.96
TICAM2 (TRAM)	Toll-like receptor adaptor molecule 2	0.84	**0.62**
TIRAP	Toll-interleukin 1 receptor (TIR)	1.09	1.00

	domain containing adaptor protein		
TOLLIP	Toll interacting protein	1.22	0.97

(O) Effectors		Regorafenib (vs. Lenvatinib) (Fold Change)	
Symbol	Name	Huh7	HepG2
CASP8 (FLICE)	Caspase 8, apoptosis-related cysteine peptide	**0.80**	0.70
EIF2AK2 (PKR)	Eukaryotic translation initiation factor 2-alpha kinase 2	1.11	**0.69**
FADD	Fas (TNFRSF6)-associated via death domain	0.81	0.87
IRAK1	Interleukin-1 receptor-associated kinase 1	1.05	1.12
IRAK2	Interleukin-1 receptor-associated kinase 2	0.75	0.76
IRAK4	Interleukin-1 receptor-associated kinase 4	1.27	0.86
MAP3K7 (TAK1)	Mitogen-activated protein kinase kinase kinase 7	0.93	0.80
TAB1	TGF-beta activated kinase 1/MAP3K7 binding protein 1	1.20	0.93
NR2C2	Nuclear receptor subfamily 2, group C, member 2	1.12	**0.64**
PPARA	Peroxisome proliferator-activated receptor alpha	1.32	1.03
PRKRA	Protein kinase, interferon-inducible double stranded RNA dependent activator	1.07	0.86
ECSIT (SITPEC)	ECSIT homolog (Drosophila)	1.16	0.83
TRAF6	TNF receptor-associated factor 6, E3 ubiquitin protein ligase	**1.22**	**0.61**

UBE2N	Ubiquitin-conjugating enzyme E2N	**0.77**	0.76
